# Effect of High-Fat Diet on the Formation of Pulmonary Neutrophil Extracellular Traps during Influenza Pneumonia in BALB/c Mice

**DOI:** 10.3389/fimmu.2016.00289

**Published:** 2016-08-02

**Authors:** Anandi Narayana Moorthy, Kong Bing Tan, Shi Wang, Teluguakula Narasaraju, Vincent T. Chow

**Affiliations:** ^1^Department of Microbiology and Immunology, School of Medicine, National University of Singapore, National University Health System, Singapore; ^2^Department of Pathology, National University Hospital, National University of Singapore, National University Health System, Singapore; ^3^Center for Veterinary Health Sciences, Oklahoma State University, Stillwater, OK, USA

**Keywords:** neutrophils, NETs, high-fat diet, influenza, pneumonia

## Abstract

Obesity is an independent risk factor for severe outcome of influenza infection. Higher dietary fat consumption has been linked to greater morbidity and severe influenza in mouse models. However, the extent of generation of neutrophil extracellular traps (NETs or NETosis) in obese individuals during influenza pneumonia is hitherto unknown. This study investigated pulmonary NETs generation in BALB/c mice fed with high-fat diet (HFD) and low-fat diet (LFD), during the course of influenza pneumonia. Clinical disease progression, histopathology, lung reactive oxygen species, and myeloperoxidase activity were also compared. Consumption of HFD over 18 weeks led to significantly higher body weight, body mass index, and adiposity in BALB/c mice compared with LFD. Lethal challenge of mice (on HFD and LFD) with influenza A/PR/8/34 (H1N1) virus led to similar body weight loss and histopathologic severity. However, NETs were formed at relatively higher levels in mice fed with HFD, despite the absence of significant difference in disease progression between HFD- and LFD-fed mice.

## Introduction

Neutrophils release neutrophil extracellular traps (NETs) to ensnare and kill pathogens, such as bacteria and fungi (1, 2), but limited studies have addressed their role in viral infections (3–5). NETs can entrap and neutralize HIV-1 in a TLR7- and TLR8-dependent manner (4). On the other hand, when NETs are present in large numbers, they can cause tissue damage by releasing cytotoxic proteins into the extracellular space. We previously revealed exuberant infiltration of activated neutrophils and extensive generation of pulmonary NETs in lethal challenge of mice with influenza virus that contribute to lung injury (6). We also demonstrated the association between pulmonary NETosis with histopathologic severity in mice during lethal pneumococcal superinfection following influenza lung infection (7, 8). These studies underscore the pathological role of pulmonary NETs during influenza pneumonia.

Obesity has been associated with chronic low-grade inflammation (9). Nutrient excess restricts blood supply to adipocytes leading to hypoxia that can incite necrosis and macrophage infiltration of adipose tissue, which in turn leads to oversecretion of pro-inflammatory cytokines (10, 11). The adipocytes themselves secrete adipokines, such as TNF-α and IL-6, that are pro-inflammatory and associated with various metabolic conditions (12, 13). Augmented peripheral neutrophil activity, such as superoxide generation, is observed in healthy obese subjects and may be attributed to cytokines, such as IL-8 secreted from the adipocytes (14, 15). Even though pro-inflammatory cytokines, such as TNF-α and IL-8, can induce NETosis (1, 16), the effects of adiposity and its associated inflammation upon NETosis are still unclear.

Obesity was documented as an independent risk factor for complications arising from severe influenza during the 2009 H1N1 pandemic (17, 18). Earlier mouse models of diet-induced obesity (DIO) have also linked the degree of obesity with severe influenza outcome and the ensuing pulmonary pathology and immune dysfunction (19–21). The association between pulmonary NETosis and the outcome of influenza pneumonia in obese subjects is hitherto unknown. Given that adipose tissues favor a pro-inflammatory environment that may potentially activate neutrophils, we hypothesize that higher adiposity can contribute to greater pulmonary NETs formation that aggravates the pathologic outcome of influenza pneumonia. The objective of this study was to investigate and compare the effects of high-fat diet (HFD) versus low-fat diet (LFD) on the extent of NETosis in the lungs of BALB/c mice during lethal influenza challenge.

## Materials and Methods

### Animals, Ethics, and Diet Regimen

All animal experiments were performed according to the regulations of the Institutional Animal Care and Use Committee, National University of Singapore (protocol number 050/11). Four-week-old male BALB/c mice were acclimatized for 1 week with standard chow before beginning a scheduled defined diet (Research Diets). Mice were randomly divided into two groups, i.e., each group was fed with either LFD (10% kcal from dietary fat) or HFD (45% kcal from dietary fat) for 18 weeks. Fresh feed was provided every week. Body weight, BMI [weight (g)/nose-to-anus length (mm^2^)], and food intake were measured weekly. The calories consumed were calculated based on food consumed (i.e., 1 g LFD = 3.85 kcal; 1 g HFD = 4.73 kcal). Blood glucose levels were measured using an Accu-Check Performa glucometer (Roche) at the start and end of the 18-week dietary schedule. At the end of the 18 weeks, organs such as lungs, brain, kidneys, heart, liver, spleen, white adipose tissue (WAT) of gonadal, perirenal regions, and interscapular brown adipose tissue (IBAT) from both groups of mice were harvested, weighed, and sectioned for hematoxylin and eosin (H&E) staining.

### Infection of Mice with Influenza Virus

Influenza virus A/Puerto Rico/8/34 (H1N1) strain (PR8) from the American Type Culture Collection was propagated in embryonated eggs, as described previously (7). After the 18-week dietary schedule, mice from both diet groups were intratracheally challenged with a lethal dose of PR8 virus, i.e., 50 plaque-forming units (PFU). Anesthesia was performed using 75 mg/kg ketamine and 1 mg/kg medetomidine, and reversed using atipamezole hydrochloride (5 mg/ml). Control mice received phosphate-buffered saline (PBS) alone. The mice were euthanized on 6 and 10 days post-infection (DPI), their lungs were excised, with one lobe fixed in 4% paraformaldehyde, while the other lobe was snap frozen for subsequent assays.

### Histopathologic Scoring of Lung Tissue

Formalin-fixed lungs were dehydrated and embedded in paraffin. Lung sections (5 μm) stained with H&E were subjected to histopathologic scoring in a blinded manner based on modified criteria (22). The final injury score was calculated according to the formula: 1 × (alveolar hemorrhage, 0–3) + 2 × (alveolar infiltrate, 0–3) + 2 × (bronchiolar infiltrate, 0–3) + 2 × (fibrin, 0–3) + 1 × (alveolar septal congestion, 0–3), where, 0–3 refer to 0 = absent, 1 = mild, 2 = moderate, and 3 = severe.

### NETs Staining and Quantification in Lung Tissue

Neutrophil extracellular traps in the lung sections were quantified by triple immunolabelling, as described previously (7). Briefly, lung sections (5 μm) were stained with antibodies against histone H2B (Abcam) and myeloperoxidase (MPO, Abcam), and DAPI (Life Technologies). NETs were identified as single strands or clusters, and scored according to pre-determined criteria (0–10). Twenty fields were analyzed, and the sum was calculated for the final NETs score.

### Determination of Viral Titers

Frozen lung tissues were homogenized using the gentleMACS tissue dissociator (Miltenyi Biotech). The viral titers were determined by plaque assay, as described previously (23).

### Hydrogen Peroxide and Myeloperoxidase Assays

Hydrogen peroxide (H_2_O_2_) concentrations in lungs were measured by the Amplex red hydrogen peroxide/peroxidase assay kit (Invitrogen). MPO activity was determined, as described previously (8). Briefly, 10 μl of lung homogenate was mixed with 190 μl of freshly prepared assay solution (26.9 ml H_2_O, 2.0 ml 0.1M sodium phosphate buffer pH 7.0, 0.1 ml 0.1M H_2_O_2_, and 0.048 ml guaiacol), and the absorbance was read immediately at 470 nm for 1 min. The MPO activity was calculated as units/ml = (ΔO.D. × *V*_t_ × 4)/(*E* × Δ_t_ × *V*_s_) × 2, where ΔO.D. = optical density change, *V*_t_ = total volume (milliliters), *E* = 26.6 mM^−1^ cm^−1^ (extinction coefficient of tetraguaiacol product), Δ_t_ = time of measurement (minutes), *V*_s_ = sample volume (milliliters), and 2 is the conversion factor to 1 cm path-length. All values were normalized to lung protein content as measured by the Bradford method (Bio-Rad).

### Statistical Analyses

Statistical analyses were performed using the SPSS (version 22). Student’s *t*-test was used for analyzing parametric data, whereas Mann–Whitney *U* test was used for non-parametric data analysis. ANOVA with Tukey *post hoc* correction was used for comparison of more than two groups. *P*-values less than 0.05, 0.01, and 0.001 were considered significant to varying extents.

## Results

### HFD Mice Gain Higher Body Weight and Body Fat Compared with LFD Mice

**Figure 1:**
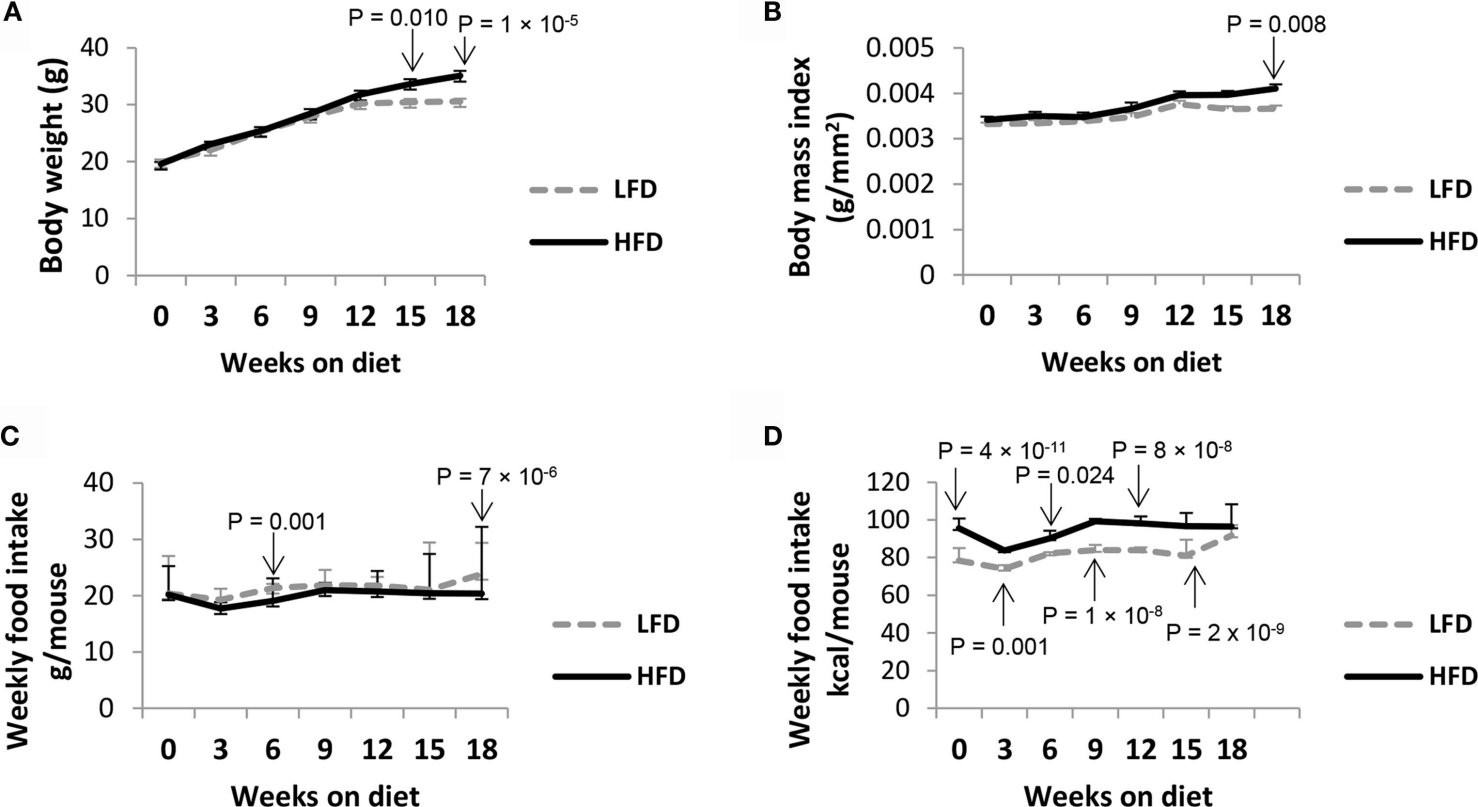
**HFD mice show higher body weight parameters compared with LFD mice**. BALB/c mice (5-week-old) were fed with either HFD or LFD for 18 weeks. Upon completion of these diets, HFD mice showed significantly higher **(A)** weight gain and **(B)** BMI. Although **(C)** the weekly intake of food per mouse was comparable between the two groups, **(D)** the actual calories consumed varied due to higher dietary fat content in HFD. Values represent the means ± SE of 15 mice per diet group (3 independent batches), by ANOVA with Tukey *post hoc* correction.

Mice on HFD showed significantly higher body weights (*P* < 0.001 on week 18) and BMI (*P* < 0.01 on week 18) compared with LFD-fed mice from 15 weeks onward, despite their weights being comparable at the start of the diet (Figures 1A,B). Although the amount of food consumed was generally similar between the groups, the HFD mice consumed more calories per mouse than the LFD mice due to higher fat percentage in HFD (*P* < 0.05 until week 15) (Figures 1C,D). However, their blood glucose levels did not reveal any significant difference (*P* = 0.239) (data not shown).

**Figure 2:**
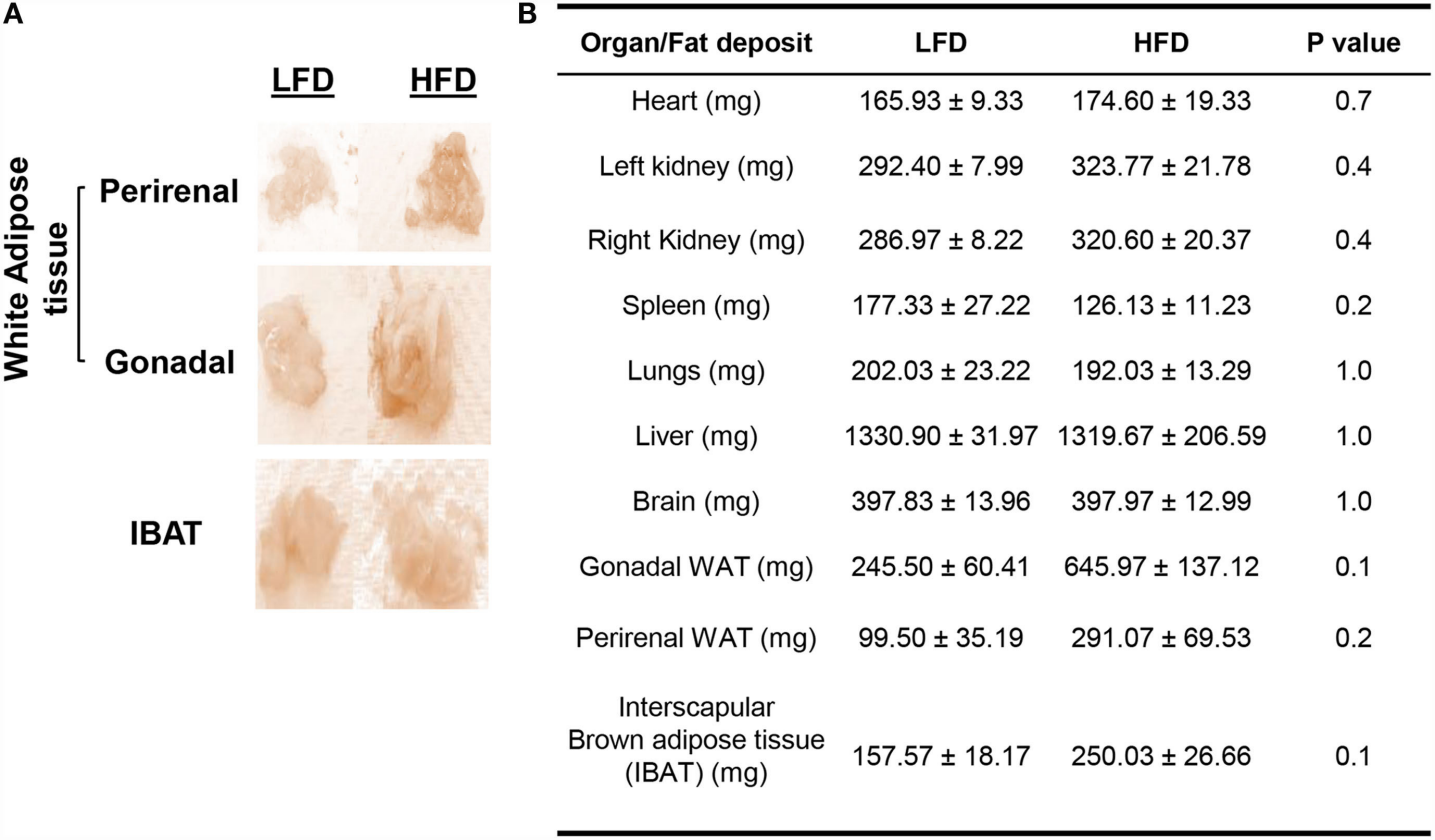
**HFD mice exhibit relatively greater body fat accumulation compared with LFD mice**. **(A)** Gross anatomy of WAT and IBAT from LFD and HFD mice. The fat pads (especially gonadal) appeared larger in the HFD group. **(B)** Wet weights of different organs and fat pads of LFD and HFD mice after their respective 18-week diet. Values represent the means ± SE of three mice per diet group (single batch), by Mann–Whitney *U* test.

The HFD mice also displayed relatively higher accumulation of WAT in the gonadal (*P* = 0.1) and perirenal fat pads (*P* = 0.2), IBAT (*P* = 0.1) (Figure 2A). Organs, such as heart (*P* = 0.7) and kidneys (*P* = 0.4), along with fat pads exhibited relatively increased weights compared with LFD mice, albeit not statistically significant (Figure 2B). The weights of spleen (*P* = 0.2), lungs (*P* = 1.0), and liver (*P* = 1.0) were slightly decreased in HFD mice, whereas the weights of brain (*P* = 1.0) were comparable between the two groups. No significant difference was observed in the histology of adipose tissue between the two groups (data not shown).

### Infected HFD Mice Have Relatively Elevated Pulmonary Viral Load but Exhibit Similar Lung Pathology as LFD Mice

**Figure 3:**
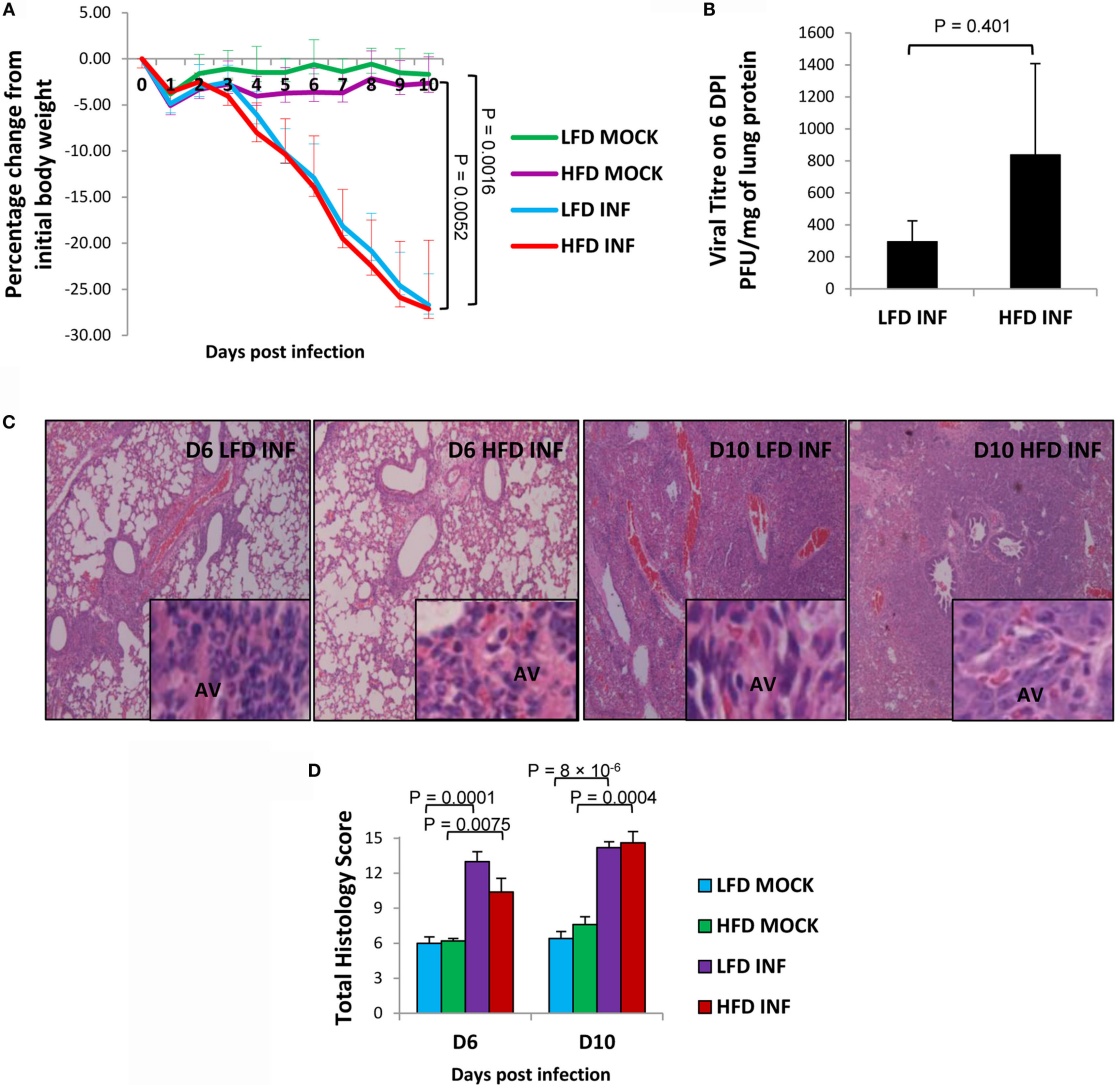
**Infected HFD mice show similar body weight loss but relatively higher lung viral titers than infected LFD mice**. Mice were infected with a lethal dose (50 PFU) of influenza A PR8 virus, and the lungs were harvested on 6 and 10 DPI. **(A)** Both groups of infected HFD (INF) and LFD (INF) mice displayed similar levels of significant body weight loss, but not control mice (MOCK) receiving PBS. **(B)** The viral titers were relatively higher in the infected HFD group at 6 DPI. No virus was detected at 10 DPI. **(C,D)** Histopathologic severity scores between the infected groups were not significantly different. AV, alveoli. Magnification: panels = 100×, insets = 1000×. Values represent the means ± SE of five mice per group (two independent experiments). Student’s *t*-test was employed for viral titer determination and ANOVA with Tukey *post hoc* correction for body weight and histopathologic analyses.

Upon lethal challenge with influenza A virus, both LFD and HFD mice showed similar body weight loss (*P* < 0.01 of both infected groups versus controls) (Figure 3A). The lung viral titers were almost threefold higher in the HFD group than LFD group, albeit not statistically significant (*P* = 0.401) (Figure 3B). A previous DIO study also observed somewhat elevated influenza viral titers in HFD mice, indicating that obesity exerts only marginal influence on viral replication within the host (19). Although histopathologic analyses demonstrated a relative reduction of overall severity score in the infected HFD group at 6 DPI (*P* = 0.108), the score became comparable with the infected LFD group by 10 DPI (*P* = 0.725) (Figures 3C,D). Both groups of mice showed thickening of alveolar septae, enhanced inflammatory cellular infiltration in the alveolar and bronchiolar spaces, and by 10 DPI, increased alveolar fibrin deposition.

### Relatively Enhanced Formation of Lung ROS and NETs in Infected HFD Mice

**Figure 4:**
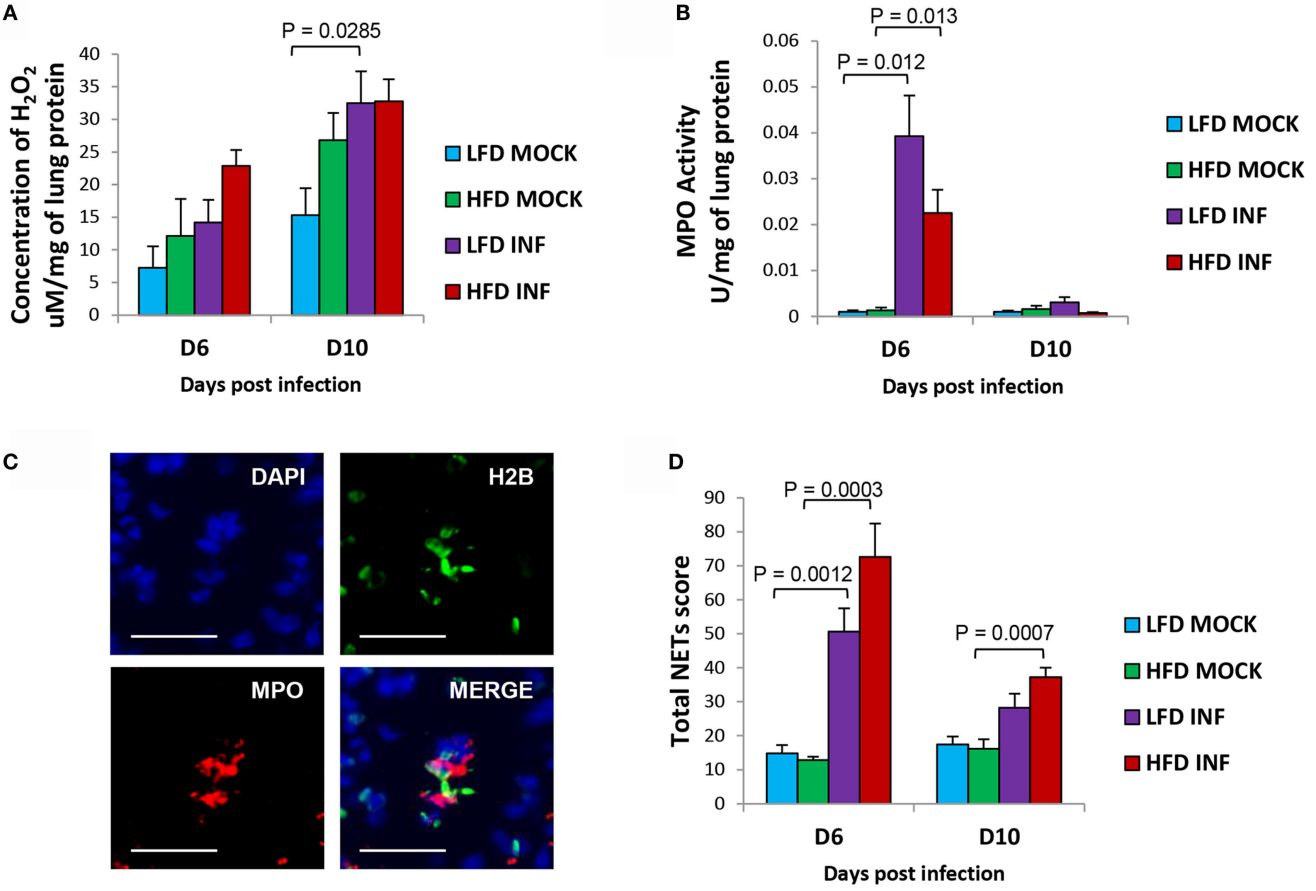
**Infected HFD mice reveal relatively higher pulmonary NETs score and H_2_O_2_ concentration**. **(A)** The concentration of H_2_O_2_ in the lungs of infected HFD mice was comparatively higher at 6 DPI. By 10 DPI, the concentrations in both infected groups were similar. **(B)** The MPO activity in the lungs of infected LFD mice was relatively higher at 6 DPI. By 10 DPI, the MPO activity was generally low in both LFD and HFD groups. **(C)** Representative images of triple immunolabeled lung tissue for identifying NETs (blue = DAPI, green = histone H2B, and red = MPO), scale bar = 25 μm. **(D)** The lungs of infected HFD mice portrayed relatively greater formation of NETs compared with LFD mice at both 6 and 10 DPI. Values represent the means ± SE of five mice per group (two independent experiments). ANOVA with Tukey *post hoc* correction was used.

The H_2_O_2_ concentration in lungs was relatively higher in infected HFD mice at 6 DPI (*P* = 0.08), indicating heightened oxidative stress in the lungs compared with LFD mice (Figure 4A). However, MPO activity was relatively lower (*P* = 0.151 and 0.128; 6 and 10 DPI) in infected HFD mice compared with LFD mice (Figure 4B). The infected HFD mice showed a trend of relatively higher generation of NETs in their lungs at 6 DPI (*P* = 0.104) and 10 DPI (*P* = 0.111) (Figures 4C,D). Our findings are congruent with previous reports documenting augmented neutrophil activity in obese individuals and murine DIO models (14, 15, 24).

## Discussion

The widespread prevalence of obesity is a cause of concern for health authorities worldwide. Besides leading to a number of metabolic disorders, such as type 2 diabetes and atherosclerosis, obesity can impact respiratory health as well (25). Obesity escalates susceptibility to influenza-related complications and community-acquired pneumonia (17, 26). While earlier studies on DIO and influenza concentrated mostly on the functions of immune cells, such as T-cells, macrophages, and neutrophils, there are hitherto no reports on the extent of NETs generated in obese individuals. Since NETs are implicated in many pathologic conditions, their importance in obesity, especially during influenza pneumonia warrants closer attention (6, 7, 27, 28).

Although our study revealed that both groups of BALB/c mice on HFD and LFD suffered similar clinical progression during influenza pneumonia, there may be subtle differences in their lung pathophysiology and immune response. We assessed and compared viral titer, H_2_O_2_ concentration, MPO activity, histopathologic severity, and formation of pulmonary NETs between the two dietary groups. If HFD consumption alters the immune response in mice, it may influence viral replication. Higher pulmonary viral load may culminate in enhanced NETs stimulation due to elevated levels of pro-inflammatory cytokine signaling and oxidative stress, given that NETosis is an oxidative process.

Generally, pulmonary viral burden, ROS concentration, and NETs formation were somewhat elevated in infected HFD mice compared with their LFD counterparts. This alludes to the subtle influences exerted by higher adiposity on these pathologic parameters. However, MPO activity was found to be slightly lower in infected HFD mice than LFD mice at 6 DPI. Similarly, the lung pathology scores were also relatively lower in HFD mice at 6 DPI. The relatively lower reduced lung viral load in infected leaner LFD mice implies a more functionally robust neutrophil response and inflammation to control the viral infection. However, we previously demonstrated that infection of neutrophils by influenza virus alone does not support active viral replication *in vitro* (29). Additional studies are thus justified to determine, if obesity and NETs release modulate virus replication and/clearance over the course of influenza pneumonia. Despite lower MPO activity, we also observed relatively greater formation of NETs in the lungs of infected HFD mice compared with infected LFD mice. Further detailed research is necessary given the involvement of MPO during NETosis, although it is still debatable whether MPO needs to be enzymatically active in this process. Contradictory reports suggest that the activity of MPO may either be non-essential during NETs stimulation (30) or is required along with its substrate H_2_O_2_ for antimicrobial activity of NETs (31).

In this study, we used BALB/c mice for consistency and comparison with our previous models on NETs during influenza pneumonia. Although the BALB/c mice on HFD were not considered strictly “obese,” they displayed sufficiently greater amount of adipose tissue to result in significant difference in BMI and body weight, which are also parameters employed in human studies (17, 18). However, other mouse strains (e.g., C57/BL6, Swiss albino mice) on HFD should also be tested for their degree of NETs generation following influenza challenge. Our study revealed a generally higher trend of pulmonary NETosis associated with adiposity. This suggests that in morbidly obese individuals, pulmonary NETs may form at significant levels in response to influenza infection that may exacerbate lung injury and complications of influenza pneumonia. In conclusion, we demonstrate that increased adiposity due to prolonged consumption of HFD may lead to relatively greater formation of NETs in murine lungs during severe influenza pneumonia.

## Ethics Statement

Experiments were performed after obtaining approval for all procedures to be performed on the animals, from the Institutional Animal Care and Use Committee, National University of Singapore (protocol number 050/11).

## Author Contributions

AM designed and performed the experiments, analyzed the data, and wrote the manuscript. SW and KT contributed to histopathologic scoring. TN contributed to data analyses. VC conceived and supervised the experiments, and wrote the manuscript.

## Conflict of Interest Statement

The authors declare that the research was conducted in the absence of any commercial or financial relationships that could be construed as a potential conflict of interest.
